# Improved rates of postoperative ischemia, completeness of aneurysm occlusion and neurological deficits in elective clipping of anterior circulation aneurysms over the past 20 years – association with technical improvements

**DOI:** 10.1007/s00701-024-06150-7

**Published:** 2024-06-07

**Authors:** Sebastian Siller, Josef Briegel, Mathias Kunz, Thomas Liebig, Robert Forbrig, Joerg-Christian Tonn, Christian Schichor, Jun Thorsteinsdottir

**Affiliations:** 1 Department of Neurosurgery, University Hospital, Ludwig-Maximilians-University of Munich (LMU), Campus Grosshadern, Marchioninistrasse 15, 81377 Munich, Germany; 2 Department of Neurosurgery, University Hospital, University of Regensburg, Franz-Josef-Strauss-Allee 11, D-93053 Regensburg, Germany; 3 Department of Anesthesiology, University Hospital, Ludwig-Maximilians-University of Munich (LMU), Campus Grosshadern, Marchioninistrasse 15, 81377 Munich, Germany; 4 Department of Neuroradiology, University Hospital, Ludwig-Maximilians-University of Munich (LMU), Campus Grosshadern, Marchioninistrasse 15, 81377 Munich, Germany

**Keywords:** Intracranial aneurysm, Anterior circulation, Clip occlusion, Outcome, Occlusion rate, Postoperative ischemic events

## Abstract

**Background/Purpose:**

Several periprocedural adjuncts for elective surgical aneurysm treatment have been introduced over the last 20 years to increase safety and efficacy. Besides the introduction of IONM in the late-1990s, ICG-videoangiography (ICG-VAG) since the mid-2000s and intraoperative CT-angiography/-perfusion (iCT-A/-P) since the mid-2010s are available. We aimed to clarify whether the introduction of ICG-VAG and iCT-A/-P resulted in our department in a stepwise improvement in the rate of radiologically detected postoperative ischemia, complete aneurysm occlusion and postoperative new deficits.

**Methods:**

Patients undergoing microsurgical clip occlusion for unruptured anterior circulation aneurysms between 2000 and 2019 were included, with ICG-VAG since 2009 and iCT-A/-P (for selected cases) since 2016. Baseline characteristics and treatment-related morbidity/outcome focusing on differences between the three distinct cohorts (cohort-I: pre-ICG-VAG-era, cohort-II: ICG-VAG-era, cohort-III: ICG-VAG&iCT-A/-P-era) were analyzed.

**Results:**

1391 patients were enrolled (*n* = 74 were excluded), 779 patients were interventionally treated, 538 patients were surgically clipped by a specialized vascular team (cohort-I *n* = 167, cohort-II *n* = 284, cohort-III *n* = 87). Aneurysm size was larger in cohort-I (8.9 vs. 7.5/6.8 mm; *p* < 0.01) without differences concerning age (mean:55years), gender distribution (m: f = 1:2.6) and aneurysm location (MCA:61%, ICA:18%, ACA/AcomA:21%). There was a stepwise improvement in the rate of radiologically detected postoperative ischemia (16.2vs.12.0vs.8.0%; *p* = 0.161), complete aneurysm occlusion (68.3vs.83.6vs.91.0%; *p* < 0.01) and postoperative new deficits (10.8vs.7.7vs.5.7%; *p* = 0.335) from cohort-I to -III. After a mean follow-up of 12months, a median modified Rankin scale of 0 was achieved in all cohorts.

**Discussion:**

Associated with periprocedural technical achievements, surgical outcome in elective anterior circulation aneurysm surgery has improved in our service during the past 20 years.

## Introduction

The treatment decision for elective intracranial aneurysm occlusion is a careful balance between the natural history of the intracranial aneurysm and the risk of the intervention based on aneurysm- and patient-specific factors. In large randomized trials, both the microsurgical clip occlusion and the endovascular coil embolization are well-established treatment modalities in ruptured [21, 33] and unruptured [5, 7] aneurysms. Especially for unruptured anterior circulation aneurysms, both clipping and coiling were demonstrated to be safe and feasible treatment options with clipping being advantageous regarding durability of long-term aneurysm repair [20].

In the last 20 years, several procedural refinements and periprocedural technical advances have been established for microsurgical clip occlusion in addition to ‘traditional’ intraoperative adjuncts (e.g. intraoperative microvascular Doppler ultrasonography). Implementation of intraoperative neurophysiological monitoring (IONM) with somatosensory and motor evoked potentials (SSEPS and MEPs) in the late 1980s and beginning 1990s can be seen as a first attempt to prevent ischemia during aneurysm surgery by monitoring brain cortical and subcortical function [9, 29]. However, introduction of the following two intraoperative imaging techniques have to be considered as the most important milestones in this regard: firstly, this is indocyanine green videoangiography (ICG-VAG) which was described in the late 1990s to indicate intraoperatively the completeness of aneurysm occlusion or to enable clip reposition in case of a stenosis [38]; the importance of ICG-VAG for an improved rate of complete aneurysm occlusion has been reported in several studies during the mid-2000s [8, 25, 26, 36]. Secondly, this is intraoperative CT-angiography/-perfusion (iCT-A/-P) which was firstly shown in the mid-2010s to be feasible in elective aneurysm surgery and to be helpful in the assessment of local and regional blood flow and cerebral perfusion immediately after clipping of intracranial aneurysms [27, 28, 36]. The above mentioned technical achievements were accompanied by improved anesthesiologic and standardized OR-procedures [1]. Along with the technical advances in microsurgical clipping, the endovascular procedures gained increasing importance and number of cases in the last decades.

Although the benefit of the above mentioned technical adjuncts seems intuitively obvious, this has never been shown in a more systematic way. Hence, we performed this retrospective study to clarify whether the introduction of ICG-VAG and iCT-A/-P resulted in our department in a stepwise improvement in the rate of

- radiologically detected postoperative ischemia,
- postoperative new deficits, and
- complete aneurysm occlusion.

For best possible conditions regarding comparability, we focussed on elective surgical repair of unruptured intracranial aneurysms (UIA) of the anterior circulation during the last 20 years.

## Methods and materials

### Patients

**Fig. 1:**
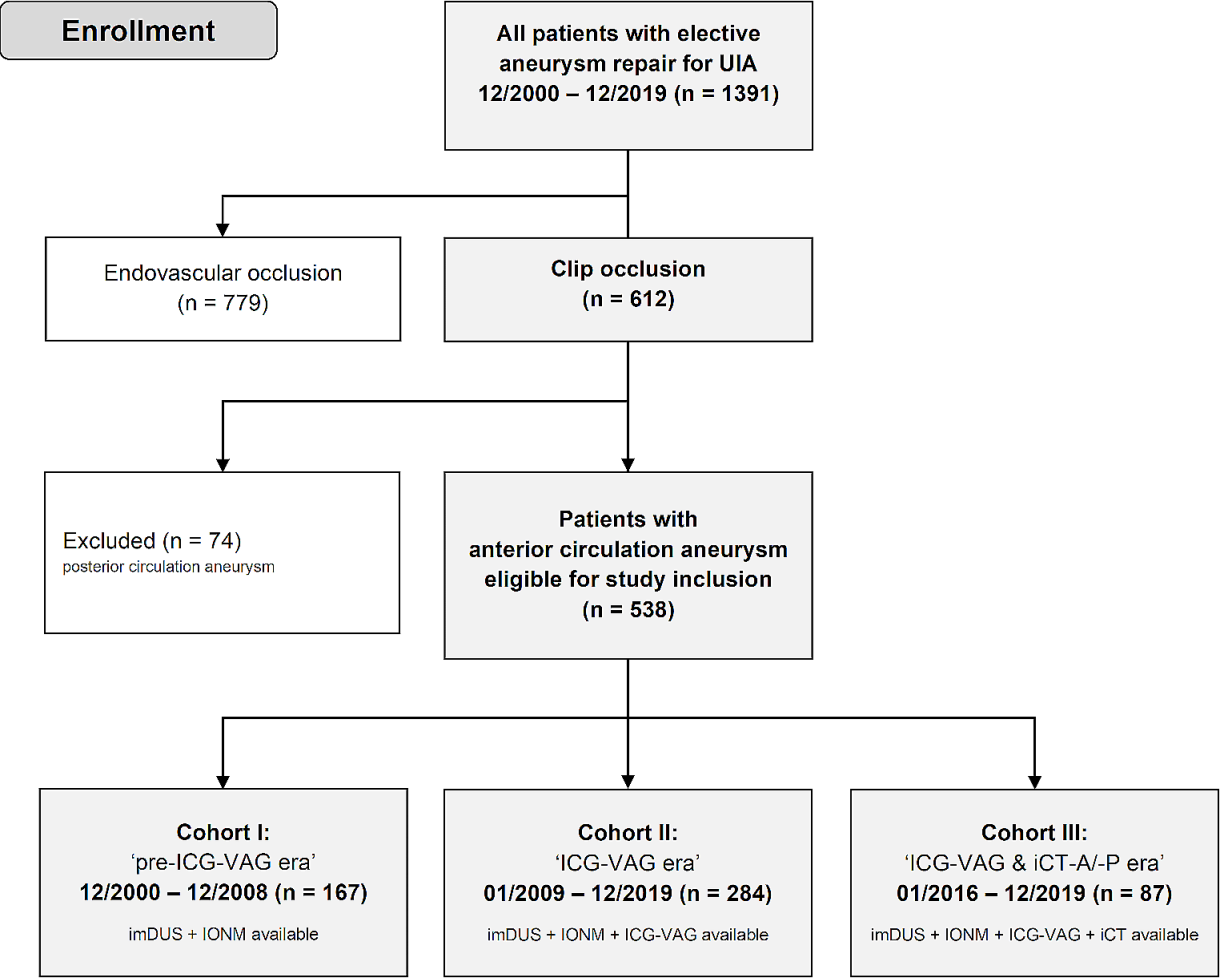
Flow-chart of patients’ inclusion for analysis. UIA: unruptured intracranial aneurysm, IONM: intraoperative neurophysiological monitoring, imDUS: intraoperative microvascular Doppler ultrasonography, ICG-VAG: Indocyaningreen-videoangiography, iCT-A/-P: intraoperative CT-angiography/-perfusion

In this retrospective study, all patients between December 2000 and December 2019 with elective microsurgical clipping of unruptured intracranial aneurysms of the anterior circulation were included. Intraoperative neurophysiological monitoring (IONM) was introduced in our department in 1999 and broadly available since the end of 2000, which marks the beginning of our series. ICG-videoangiography (ICG-VAG) was introduced and broadly available since 2009 and intraoperative CT-angiography/-perfusion (iCT-A/-P) since 2016. Accordingly, we divided our patient population in a ‘pre-ICG-VAG’ era vs. ‘ICG-VAG era’ vs. ‘ICG-VAG & iCT-A/-P era’. Accordingly, cohort I included patients between December 2000 and December 2008 (‘pre-ICG-VAG era’), cohort II included patients between January 2009 and December 2019 (‘ICG-VAG era’), and cohort III included patients between January 2016 and December 2019 which were eligible for iCT-A/-P in addition to ICG-VAG (‘ICG-VAG & iCT-A/-P era’). See also flow-chart of patients’ inclusion for analysis in Fig. 1.

Microvascular Doppler ultrasonography was intraoperatively available during all three above mentioned eras. Throughout the whole observation period (2000–2019), indication for microsurgical clip occlusion was based on the recommendations of our interdisciplinary neurovascular board (INVB) in accordance with evidence-based guidelines for UIA treatment of national and international specialist societies at the respective time periods [31]. Microsurgical clipping was performed by a specialized neurovascular team consisting of three staff neurosurgeons specialized in neuro-vascular microsurgery. Although the members of that group changed over time (but not in parallel to the above mentioned cohorts/treatment eras), the qualification within the neurovascular team remained similar and part of that group remained the same.

Patient characteristics included age, sex, and neurological status. Aneurysm-specific characteristics comprehended side, location, size and temporary clipping. We reviewed operative records and analyzed pre-/post-operative angiography and subsequent imaging data (CT and/or MRI) during in-hospital stay in all cases as well as IONM, ICG-VAG and iCT (including unenhanced brain CT, CT-A and CT-P as described elsewhere [36]) data whenever applied. Clinical outcome was assigned by the Modified Rankin Scale (MRS) postoperatively, at discharge and subsequent follow-up (FU) visits. The study was approved by the institutional review board (No.19–560).

### Clinical protocol

Baseline characteristics and treatment-related morbidity/outcome were analyzed according to the three distinct cohorts with special regard to detection of postoperative new neurological deficits, ischemic events and aneurysm occlusion rates.

Before induction of general anesthesia, invasive arterial blood pressure measurement was established and performed via radial or femoral arterial lines. General anesthesia was induced intravenously with a bolus of propofol (2-3 mg/kg), sufentanil (30 μg) and additionally 70 μg for Mayfield clamp fixation. A medium acting muscle relaxant (cisatracurium, 0.1 mg/kg bolus) was administered for intubation purposes only. To enable IONM, total intravenous anesthesia was chosen and maintained with propofol (5–8 mg/kg/hr) and remifentanil (0.3–0.5 μg/kg/min). Mean arterial pressure (MAP) was kept within a narrow corridor of 70 to 85 mmHg during induction and maintenance of anesthesia. When the MAP fell below 70 mmHg, low-dose norepinephrine was administered and adjusted intravenously via a syringe pump. Elevated blood pressure was treated either intravenously with urapidil (5 to 10 mg) or with short courses of inhaled sevoflurane (0.5 to 1.0 MAC) in consultation with the neurosurgeon and the team that performed the IONM.

### Surgery

During surgery, after aneurysm exposure, parent and branching arteries were identified. In most cases a microvascular Doppler ultrasonography (Vascular Doppler Systems, Mizuho, Tokyo, Japan) was used before and after clip ligation (Sugita titanium aneurysm clips, Mizuho, Tokyo, Japan or Yasargil Clips, Peter Lazic GmbH, Tuttlingen, Germany). Since 2000, intraoperative neurophysiological monitoring (IONM), including MEP (muscle evoked potential) and SEP (sensory evoked potential), was broadly available in our department to be performed during surgery in case it was deemed to be necessary by the lead vascular neurosurgeon for the individual case. In case of pathological events, the neurosurgeon was informed immediately. Since 2009, if clip positioning deemed satisfactory, ICG-VAG was performed and the video analysis was then correlated with the findings from visual inspection and parent artery Doppler ultrasonography. After ICG-VAG application, in case of a clip stenosis or aneurysm remnant, the clip was inspected and repositioned if necessary. Since 2016, intraoperative unenhanced CT was additionally performed in selected patients to rule out ischemia, edema-formation or hematoma, while iCT-A was performed to detect major incomplete Clip-occlusion or discontinuation of the branching arteries. ICT-P was performed to rule out perfusion deficits unveiling impeding ischemia.

### Intraoperative neurophysiological monitoring (since 2000)

IONM was similarly performed in all three distinct cohorts upon availability of the neuromonitoring team or specific request of the vascular neurosurgeon as previously described [34, 35]. IONM was deemed to be reasonable in case of all types of internal carotid artery and anterior cerebral artery (incl. anterior communicating artery) aneurysms as well as proximal middle cerebral artery aneurysms, while IONM as deemed to be facultative for distal middle cerebral artery aneurysms. Warning was given if three consecutive MEP showed a marked isolated increase of stimulation intensity > 20% for the respective hemisphere and amplitude decrement > 80% and/or cortical SEP-amplitudes showed an amplitude decrement > 50%.

### Indocyanine green videoangiography (since 2009)

Operations were performed using a microscope-integrated infra-red sensitive monochrome video camera (OPMI Pentero with INFRA-RED800, Zeiss, Oberkochen, Germany). Fluorescent dye (indocyanin green, Verdy^®^; Diagnostic Green GmbH, Aschheim-Dornach, Germany) was administered intravenously (10 mg per dose, 0.2–0.5 mg/kg body weight) as described previously [24]. Images were continuously displayed to evaluate visualisation quality and initial dye inflow. Real-time flow in arteries, branching vessels and perforators was observed as well as flow analyses were performed (incl. analysis of transit times by FLOW 800 in selected cases). In case of irregularities, the clip was repositioned or additional clips were used. Repeated flow analyses were performed until the clipping result was sufficient.

### Intraoperative computed tomography angiography and perfusion (since 2016)

Since its introduction in our institute, iCT (incl. unenhanced brain CT, CT angiography and CT perfusion) was used in addition to ICG-VAG (cohort III) upon availability, patient’s consent for iCT use and exclusion of contraindications (e.g. iodine allergy or renal insufficiency). Patients that were treated since 2016 but were not eligible for iCT were allocated into cohort II. ICT was performed using Siemens SOMATOM Definition AS+, Siemens Healthineers (Siemens AG, Munich, Germany) with technical/radiological details as recently described [36]. Subsequently, CT and automated contrast agent/saline injection were performed within 90–120 s. 3D postprocessing of iCT angiography and dynamic perfusion analysis of iCT perfusion data set was achieved within 3 min. by a technical assistant. During scanning, the surgical field was secured by a sterile drape. After data acquisition/reconstruction, the neuroradiologist reviewed the iCT and immediately reported any pathologic findings to the neurosurgeon. Regarding CT angiography, irregularity of the reconstructed parent and branching vessels were identified as a hint for an aneurysm remnant and clip reposition was performed on discretion of the performing surgeon. Regarding CT perfusion, a mismatch was defined as prolonged mean transit time with moderate reduction of the cerebral blood flow and normal/increased cerebral blood volume. According to the iCT findings, in case of a mismatch the operative field was reinspected and the clip was repositioned when an obvious stenosis could be detected. In all other cases conservative treatment e.g. elevation of mean arterial pressure, application of anticonvulsants, nimodipine, and/or anticoagulation was applied.

### Radiological and clinical assessment

A radiologically detected ischemia was defined as a newly detected cerebral hypodensity in the postoperative CT scan. A radiologically detected aneurysm remnant was rated if the postoperative CT angiography or DSA showed an irregularity of the reconstructed parent and branching vessels. A postoperative deficit was classified as any new neurological deterioration including cognitive impairment, sensorimotor deficit, aphasia or seizure.

### Statistical analysis

Continuously or ordinally scaled variables were analyzed with Mann-Whitney U test or Kruskal-Wallis One Way Analysis of Variance on Ranks (ANOVA), categorical variables with Chi-square or Fisher’s exact test. Factors associated with surgical outcome (aneurysm remnant, postoperative ischemia) and unfavorable neurological outcome at last FU were identified using univariate analysis. Significant variables were included in multivariate logistic regression analysis with determination of Odds ratio (OR) and 95%-confidence interval (95%-CI). A *p* value ≤ 0.05 was considered significant. All calculations were performed using Sigma Plot for Windows v.11 (Systat Software Inc., San Jose, California, United States).

## Results

### Baseline characteristics

**Fig. 2:**
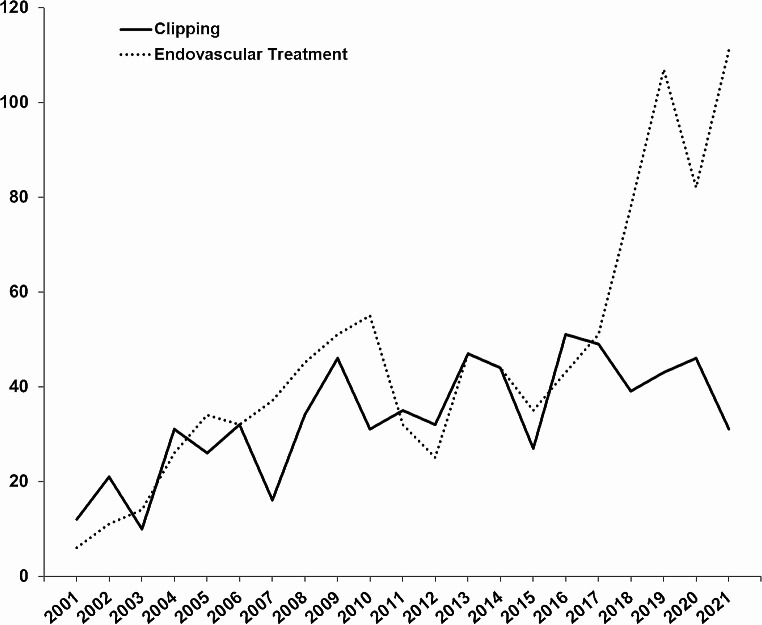
Annual numbers of patients treated for elective microsurgical and endovascular repair of unruptured intracranial aneurysma between December 2001and December 2022

Numbers of patients treated in our department since December 2000 for UIA for either treatment modality are displayed over time in Fig. 2. There was an increase in treatment numbers for both treatment modalities over time as well as a shift from a microsurgical-dominated treatment pattern to an endovascular-dominated treatment pattern especially in the last five years.

For our study, we in detail reviewed the data of 612 patients undergoing microsurgical elective UIA repair during December 2000 and December 2019 in our department. While 74 patients with posterior circulation aneurysms (incl. aneurysms of the posterior communicating artery) were excluded, 538 patients with elective microsurgical clip occlusion of an anterior circulation UIA during our 20-years observational period were eligible for study inclusion (see Fig. 1). Mean age was 55 ± 11 years and there was a predominance for the female gender (female/male: 2.6:1). Most frequent aneurysm location was the middle cerebral artery (61%) followed by the internal carotid artery (18%) and anterior cerebral artery incl. anterior communicating artery (21%).

Among these 538 patients, cohort I (1999–2008, ‘pre-ICG-VAG era’) included 167 patients, cohort II (2009–2019, ‘ICG-VAG era’) included 284 patients (among those 95 patients treated between 2016 and 2019) and cohort III (2016–2019, ‘ICG-VAG & iCT-A/-P era’) included 87 patients. Baseline characteristics of patients separated for the three distinct cohorts are displayed in Table 1 and did not significantly differ. However, patients had a slightly smaller mean aneurysm size (7.5 and 6.8 vs. 8.9 mm) in the more recent cohorts II and III compared to cohort I.

**Table 1:** Baseline characteristics

Characteristics	Patients with clip occlusion for UIA in the anterior circulation			*p*-value
	cohort I 1999–2008 (*n* = 167)	cohort II 2009–2019 (*n* = 284)	cohort III 2016–2019 (*n* = 87)	
gender, male/female	35 / 132	87 / 197	28 / 59	0.835
mean age, yrs ± SD	53 ± 10	57 ± 11	55 ± 12	0.103
aneurysm location, no. (%)				
ICA	43 (26%)	39 (14%)	15 (17%)	0.128
MCA	97 (58%)	184 (65%)	47 (54%)	0.591
ACA and AcomA	27 (16%)	61 (21%)	25 (29%)	0.142
mean aneurysm size, mm ± SD	8.9 ± 4.8	7.5 ± 3.7	6.8 ± 3.0	< 0.001 (I vs. II/III)

UIA: unruptured intracranial aneurysm; ICA: internal carotid artery; MCA: middle cerebral artery; ACA: anterior cerebral artery; AcomA: anterior communicating artery

Mean values ± standard deviation (SD)

### Intraoperative imaging findings and surgical management

In cohort-II, clip relocation was performed in 13/284 patients (4.6%) due to ICG-VAG results revealing a clip stenosis or an aneurysm remnant. In cohort III (receiving ICG-VAG and iCT-A/-P), according to a mismatch in intraoperative CT perfusion due to a clip stenosis, clip relocation was performed in 2/84 patients (2.4%). In detail, a 61 years old male patient harboring a calcified 26 mm MCA aneurysm had a mismatch in the frontal operculum in iCT-P with immediate intraoperative clip repositioning, while iCTP showed a mismatch in both anterior territories in a 71 years old female patient harboring a 7 mm Acom aneurysm with following intraoperative clip repositioning. Furthermore, due to mismatches in the iCT-P, an intensified conservative treatment was intraoperatively applied in 5/84 patients (6.0%). Also, clinically relevant aneurysm remnants were detected in iCT angiography in 2/84 patients (2.4%) leading to immediate clip relocation. In detail, a 53 years old male patient with a 11 mm Acom aneurysm showed a clinically relevant aneurysm remnant in iCT-A leading to successful intraoperative clip repositioning; a 51 years old female patient harboring a 10 mm Acom aneurysm showed a clinically relevant aneurysm remnant in iCT-A, as clip repositioning was not successful intraoperatively, this patient was reoperated from the contralateral side.

### Postoperative ischemia events

The rate of patients with radiologically detected postoperative ischemia was 12.6%in the overall patient population. While the rate of patients with radiologically detected postoperative ischemia was 16.2% in cohort I, it improved to 12.0% in cohort II and 8.0% in cohort III, although significance was not reached for the differences between the three cohorts when comparing the analysis of variance on ranks (*p* = 0.161, Table 2).

**Table 2:** Outcome parameters

Characteristics	Patients with clip occlusion for UIA in the anterior circulation			*p*-value
	cohort I 1999–2008 (*n* = 167)	cohort II 2009–2019 (*n* = 284)	cohort III 2016–2019 (*n* = 87)	
rate of radiologically detected postoperative ischemia, %	16.2	12.0	8.0	0.161
rate of radiologically detected aneurysm occlusion, %	68.3	83.6	91.0	< 0.001 (I vs. III)
rate of postoperatively new or aggravated deficits, %	10.8	7.7	5.7	0.335

### Postoperative neurological deficits and outcome

The overall rate of postoperative new or aggravated neurological deficits was 8.4%. The rate of postoperative new or aggravated neurological deficits was highest in cohort I with 10.8% and was reduced to 7.7% and 5.7% in cohort II and III, even though statistical significance was also not seen (*p* = 0.335, Table 2). Median MRS Score at last follow-up (mean: 12months) was 0 in all three cohorts without significant differences.

### Aneurysm occlusion rates

In the overall patient population, the rate of radiologically confirmed complete aneurysm occlusion was 81.7%. The rate of radiologically confirmed complete aneurysm occlusion markedly improved from 68.3% in cohort I to 83.6% in cohort II and as high as 91.0% in cohort III with significance being reached when comparing cohort I and III (*p* < 0.001, Table 2).

### Risk factor analysis

Both aneurysm size as well as aneurysm location were significant risk factors for all three outcome parameters in uni- and multivariate analysis, while treatment in the ‘pre-ICG-VAG era’ (cohort I vs. cohort II/III) was a significant risk factor in uni- and multivariate analysis for a higher rate of radiologically detected incomplete aneurysm occlusion. The strongest risk factor for new or aggravated postoperative deficits was the rate of detected postoperative ischemia in both uni- and multivariate analysis (Table 3).

**Table 3:** Risk factors

	rate of radiologically detected postoperative ischemia OR (*p* value / 95% CI)	rate of radiologically detected incomplete aneurysm occlusion OR (*p* value / 95% CI)	rate of postoperatively new or aggravated deficits OR (*p* value / 95% CI)
**Univariate**			
**gender** male vs. female	0.64 (0.15 / 0.34–1.18)	0.65 (0.16 / 0.35–1.19)	0.72 (0.38 / 0.35–1.49)
**age (years)** per year	1.01 (0.47 / 0.99–1.03)	1.00 (0.70 / 0.97–1.02)	1.00 (0.95 / 0.97–1.03)
**aneurysm location** ICA vs. MCA vs. AcomA	1.39 (0.03 / 1.04–1.87)	1.36 (0.05 / 1.00-1.85)	1.58 (< 0.01 / 1.12–2.23)
**aneurysm size (mm)** per mm	1.09 (< 0.01 / 1.03–1.15)	1.11 (< 0.01 / 1.04–1.18)	1.09 (< 0.01 / 1.03–1.16)
**radiologically detected postoperative ischemia** yes vs. no	/	/	23.95 (< 0.01 / 11.86–48.35)
**treatment era** cohort I vs. II vs. III	0.68 (0.06 / 0.46–1.01)	0.45 (< 0.01 / 0.29–0.69)	0.71 (0.15 / 0.44–1.13)
**Multivariate**			
**gender** male vs. female	0.60 (0.13 / 0.31–1.17)	0.61 (0.15 / 0.31–1.2)	0.58 (0.21 / 0.25–1.35)
**age (years)** per year	1.01 (0.36 / 0.99–1.04)	0.99 (0.62 / 0.97–1.02)	1.00 (0.82 / 0.97–1.04)
**aneurysm location** ICA vs. MCA vs. AcomA	1.59 (< 0.01 / 1.16–2.19)	1.62 (< 0.01 / 1.15–2.28)	1.78 (< 0.01 / 1.20–2.64)
**aneurysm size (mm)** per mm	1.08 (< 0.01 / 1.02–1.15)	1.10 (< 0.01 / 1.03–1.18)	1.10 (< 0.01 / 1.02–1.16)
**radiologically detected postoperative ischemia** yes vs. no	/	/	21.12 (< 0.01 / 9.71–45.93)
**treatment era** cohort I vs. II vs. III	0.68 (0.08 / 0.44–1.05)	0.53 (< 0.01 / 0.34–0.84)	0.67 (0.14 / 0.39–1.14)

## Discussion

This retrospective study was performed to collect data of elective surgical repair of UIA during the last two decades in consecutive distinct cohorts of the ‘pre-ICG-VAG era’, ‘ICG-VAG era’ and ‘ICG-VAG & iCT-A/-P era’ in our department and compare for changes in the rate of (i) radiologically detected postoperative ischemia, (ii) postoperative new deficits and, (iii) complete aneurysm occlusion. We could show a reduced rate of radiologically detected postoperative ischemia (16.8% vs. 12.0% vs. 8.0%), a decreased rate of patients with postoperative new/aggravated neurological deficits (10.8% vs. 7.7% vs. 5.7%), and an improved rate of radiologically confirmed complete aneurysm occlusion (68.3% vs. 83.6% vs. 91.0%) alongside to the stepwise introduction of intraoperative imaging technologies.

In the last two decades, the expansion of IONM modalities around the year 2000, the widespread use of ICG-VAG in the mid-2000s as well as the introduction of iCT-A/-P in the mid-2010s can be considered as milestones [16, 27, 28, 38]. Accompanying the mentioned technical advances, the expansion of endovascular approaches involved an obvious change in indications for vascular surgery. The proportion of surgically treated aneurysm shifted towards a higher proportion of anterior circulation aneurysms and aneurysms with a complex configuration [18, 19]. In our neurovascular center, the number of microsurgically clipped patients for UIA of the anterior circulation increased in the first decade of our observational period and remained stable over the latter decade without any significant change in aneurysm size or location between the three distinct cohorts.

While the majority of studies evaluated the impact of a single technical innovation on patients’ morbidity and mortality, studies distinctly evaluating the effect of ICG-VAG and iCT-A/-P besides IONM plus intraoperative microvascular Doppler ultrasonography on outcome parameters of surgical aneurysm repair are still lacking. Only the multicenter study of Luther et al., which investigated outcome changes in UIA surgery in the post-International Subarachnoid Aneurysm Trial (ISAT) era between 2004 and 2014, is one of few exceptions [19], even so the study comprises only part of the Continuing Innovations era and lacks changes in the past decade. It is surprising that Luther et al. could show a worsened rate of routine discharge as a surrogate parameter for a more unfavorable outcome over time in their cohort which seems to be counterintuitive considering all (peri)procedural improvements during that timeperiod [19]. In contrast, we could show a decreased rate of patients with postoperative new/aggravated neurological deficits from 10.8% in cohort I to as low as 5.7% in cohort III (compared to as low as 3.9–6.6% to as high as 21.8% in literature [2, 10, 17, 37]). Also, a favourable outcome with a MRS of 0 was achieved in all our cohorts.

We could also show a stepwise improvement in the rate of radiologically detected postoperative ischemia from 16.8 to 12.0% and 8.0% between cohort I vs. II vs. III. A comparison of contemporary literature data showed that the results of our latest treatment cohort were very similar (8.0% in our cohort compared to as low as 7.5 to as high as 13.0% in literature [14, 17, 30, 37]), indicating good data validity and reliability. We hypothesize that improved surgical and clinical outcome could be achieved by using ICG-VAG and iCT-A/-P; e.g. in cohort-II intraoperative clip relocation was performed in 4.6% due to ICG-VAG results and in cohort-III in 2.4% due to clip stenosis (and in 2.4% due to aneurysm remnant) which was detected by iCT-A/-P. This is in line with several studies from the mid-2000s that provided evidence that ICG-VAG can intraoperatively demonstrate incomplete aneurysm occlusion or clip stenosis [38]; intraoperative clip repositioning was reported in 9–15% of cases due to ICG-VAG results [8, 25, 26]. Recently, we also specifically showed that iCT-A/-P is able to detect impending ischemic events in up to 7.5% of cases and allows immediate intraoperative correction where IONM and ICG-VAG is false-negative [36]. Those aneurysm locations that benefited most of iCT-A regarding aneurysm remnants were especially observed in cases where the microscopic field was limited, e.g. in Acom aneurysms. Benefits of iCT-P regarding clip stenosis were also observed in those cases where the microscopic field was very narrow, e.g. in Acom aneurysms where ICG-VAG suggested the suspicion of discrete flow velocity differences of the distal branches but no proof of definite vessel occlusion. Also, iCT-P was advantageous in calcified and partially thrombosed aneurysms, where aneurysm neck reconstruction was challenging. The number needed to treat to prevent one case of ischemic events or one aneurysm remnant detected through the application of iCT-A/-P was statistically determined to be 25 cases each.

While the changes in the rates of postoperative new/aggravated neurological deficits and postoperative ischemia between cohort I and III showed a clear trend but did not reach statistical significance (probably due to the limited and imbalanced sample size), the improved rate of radiologically confirmed complete aneurysm occlusion between cohort I and III did reach significance, – With regard to incomplete aneurysm occlusion we could show a rate of 9.0% in our latest treatment cohort, which is also very similar to the rates of 5.9% up to 15.8% in literature [22, 32]. In our cohort, the association to a distinct treatment cohort was the most significant risk factor for the rate of radiologically detected incomplete aneurysm occlusion in both uni- and multivariate analysis. Unsurprisingly, the presence of radiologically detected postoperative ischemia was the strongest risk factor for a worse neurological outcome, whereas aneurysm size and location (even within the anterior circulation) were risk factors for an unfavorable surgical and clinical outcome in uni- and multivariate analysis. This is in line with previous publications showing aneurysm size and other morphological aneurysm features to be risk factors for poor outcome or ischemic events [6, 11, 15, 23].

### Limitations

One limitation of our study is the retrospective and single center design as well as the imbalanced sample size of the distinct cohorts. Furthermore, we have to state that all aneurysm remnants which were radiologically detected were rated, including minimal remnants. Also, the rate of radiologically detected postoperative ischemia could also compromise CT hypodensities due to surgical manipulation and not apparent ischemias. Moreover, since IONM was used in all three distinct cohorts with similar indications, we cannot assess the impact of IONM alone on outcome with our dataset; however, previous data from our institute suggest that the introduction of IONM does not necessarily improve overall long-term outcome in elective aneurysm clipping [12] which challenges previous data of Schramm et al. [29] and Byoun et al. [3].

Furthermore, endovascular approaches expanded in the last few years and simultaneously the proportion of surgically treated aneurysm shifted towards a higher proportion of aneurysms with a complex configuration which was mostly true for posterior circulation aneurysms, while this trend was markedly lower for anterior circulation aneurysms like MCA aneurysm as outlined in previous publications [4, 13, 37]. We could also see this development with a relative overall shift towards endovascular repair in our institute during the study period (as outlined in Fig. 2), but with a focus on anterior circulation aneurysms (excluding posterior circulation aneurysm and aneurysms of the posterior communicating artery) our dataset was not designed for a dedicated evaluation of a change in ‘complexity’ of surgically treated aneurysms over time. However, with a focus on anterior circulation aneurysms only and without any significant changes in baseline and aneurysm characteristics (except for a slightly smaller mean aneurysm size in the more recent cohorts II and III compared to cohort I) between the three distinct cohorts, we do not have any hints for a relevant ‘complexity bias’ as a confounder in our study.

Moreover, even so we compare treatment outcomes between three distinct cohorts according to the stepwise addition of intraoperative imaging innovations in our department, another limitation of our study is that it is not entirely possible to attribute outcome changes between the distinct cohorts to the introduction of a single innovation and distinguish that from general evolvements in surgical and perioperative treatment concepts or a coincidental global evolution of surgical proficiency. However, cohort II and cohort III of our study include patients treated by the same vascular team during an overlapping timeperiod – making both cohorts very comparable and robust against confounders (e.g. surgical training over time) – with the only difference being the addition of ICT-A/-P. As outlined in our previous paper [36], addition of iCT-A/-P is able to detect impending ischemic events (and allows for immediately intraoperative correction) in up to 7.5% of cases where IONM and ICG-VAG failed to demonstrate these circumstances. Furthermore, iCT-A/-P in cohort III also enabled additional detection of clinically relevant aneurysm remnants which lead to immediate intraoperative clip relocations especially in selected aneurysms with a complex configuration. The improvement in outcomes between these two cohorts therefore supports the hypothesis of a stepwise improvement of outcome in parallel with the introduction of intraoperative assisting techniques in elective UIA surgery of the anterior circulation.

## Conclusion

Here, we analysed surgical and neurological outcome in patients with microsurgical clipping of UIA of the anterior circulation being treated in three consecutive, distinct periods within two decades: (i) prior to introduction of ICG-VAG (‘pre-ICG-VAG era’), (ii) after introduction of ICG-VAG (‘ICG-VAG era’) and (iii) after introduction of iCT-A/-P (‘ICG-VAG & iCT-A/-P era’). A stepwise improvement in the rate of postoperative ischemia (16.2% vs. 12.0% vs. 8.0%), neurological deficits on the first postoperative day (10.8% vs. 7.7% vs. 5.7%) and aneurysm occlusion (68.3% vs. 83.6% vs. 91.0%) could be demonstrated. The introduction of these intraoperative imaging modalities was therefore associated with improvements of surgical and neurological outcome. Overall, median MRS Score at last follow-up was 0 in all three cohorts without significant differences. The association to an early treatment period was the most significant risk factor for the rate of radiologically detected incomplete aneurysm occlusion. The presence of radiologically newly detected postoperative ischemia was the strongest risk factor for a worse neurological outcome in multivariate analysis.

## Data Availability

The data that support the findings of this study are available from the corresponding author, Sebastian Siller, upon reasonable request.

## Abbreviations

ACA: Anterior cerebral artery
AcomA: Anterior communicating artery
ANOVA: Analysis of Variance on Ranks
ca.: Circa
CI: Confidence interval
CT: Computed tomography
FU: Follow-up
ICA: Internal carotid artery
ICG-VAG: Indocyanine green videoangiography
iCT-A/-P: Intraoperative CT-angiography/-perfusion
incl.: Including
INVB: Interdisciplinary neurovascular board
IONM: Intraoperative neurophysiological monitoring
ISAT: International Subarachnoid Aneurysm Trial
MAP: Mean arterial pressure
MCA: Middle cerebral artery
MEPs: Motor evoked potentials
MRI: Magnetic resonance imaging
MRS: Modified Rankin Scale
OR: Odds ratio
resp.: Respectively
SD: Standard deviation
SSEPs: Somatosensory evoked potentials
UIA: Unruptured intracranial aneurysms
